# Modulation of Cortical Interhemispheric Interactions by Motor Facilitation or Restraint

**DOI:** 10.1155/2014/210396

**Published:** 2014-02-24

**Authors:** Ana Cristina Vidal, Paula Banca, Augusto Gil Pascoal, Gustavo Cordeiro, João Sargento-Freitas, Miguel Castelo-Branco

**Affiliations:** ^1^University of Lisbon, Faculty of Human Kinetics, CIPER, LBMF, 1499-002 Lisbon, Portugal; ^2^Garcia de Orta Hospital, Pragal, 2801-951 Almada, Portugal; ^3^Visual Neuroscience Laboratory, Institute for Biomedical Imaging in Life Sciences (IBILI), ICNAS, Faculty of Medicine, University of Coimbra, Azinhaga de Santa Comba, 3000-548 Coimbra, Portugal; ^4^Stroke Unit, Department of Neurology, Coimbra University Hospital, 3000-075 Coimbra, Portugal

## Abstract

Cortical interhemispheric interactions in motor control are still poorly understood and it is important to clarify how these depend on inhibitory/facilitatory limb movements and motor expertise, as reflected by limb dominance. Here we addressed this problem using functional magnetic resonance imaging (fMRI) and a task involving dominant/nondominant limb mobilization in the presence/absence of contralateral limb restraint. In this way we could modulate excitation/deactivation of the contralateral hemisphere. Blocks of arm elevation were alternated with absent/present restraint of the contralateral limb in 17 participants. We found the expected activation of contralateral sensorimotor cortex and ipsilateral cerebellum during arm elevation. In addition, only the dominant arm elevation (hold period) was accompanied by deactivation of ipsilateral sensorimotor cortex, irrespective of presence/absence of contralateral restraint, although the latter increased deactivation. In contrast, the nondominant limb yielded absent deactivation and reduced area of contralateral activation upon restriction. Our results provide evidence for a difference in cortical communication during motor control (action facilitation/inhibition), depending on the “expertise” of the hemisphere that controls action (dominant versus nondominant). These results have relevant implications for the development of facilitation/inhibition strategies in neurorehabilitation, namely, in stroke, given that fMRI deactivations have recently been shown to reflect decreases in neural responses.

## 1. Introduction

The role of interhemispheric communication in motor action is still highly debated concerning the role of inhibition and facilitation [1]. Most functional magnetic resonance imaging (fMRI) studies of limb function have focused on hand motion [2–5] and very few were concerned with arm and shoulder motion [6, 7]. In spite of the lack of consensus on how interhemispheric interactions modulate motor control, it is well established that the neural circuits underlying upper limb voluntary movements include the contralateral sensorimotor cortex [8], basal ganglia [9], and the ipsilateral cerebellum (intermediate hemispheric portion of Larsell lobules H IV-V) [10].

Motor representations corresponding to inhibition/deactivation are less well documented [2, 4]. Assumptions on inhibition rely on its controversial links with negative blood oxygenation level dependent (BOLD) signal [11–13]. Two previous studies [11, 14] do nevertheless show evidence for the fact that negative BOLD reflects suppression of neuronal activity. Accordingly, Shmuel et al. showed that visual negative BOLD response is related to decreases in neuronal activity below spontaneous baseline activity, rather than a purely vascular origin [11, 15]. Liu et al. also found the suppression of neuronal activity as the origin of negative responses in other brain regions [14]. These results show that it is possible to relate BOLD deactivation at least to decreases in neural responses. This is what we mean by deactivation and its link to inhibition in this paper.

The understanding of how interhemispheric facilitation/inhibition influences action control is a basic science goal that is also relevant to the development of new approaches in neurorehabilitation. If successful, this approach might help improve the design of neuromodulation approaches such as the ones used in transcranial magnetic stimulation (TMS), which is a technique that can be used to manipulate levels of inhibition and/or facilitation in the human motor system. This idea is also connected to the excitation/inhibition theories that are well established in the physiotherapy domain. For example, restriction of undesired movements helps reducing the activation of compensatory motor patterns, some of which maybe excessive. Previous studies suggest that recruitment of compensatory movements may have a negative impact on motor recovery [16–18] like the excessive trunk movements in reaching, which attempt to compensate the lack of motion in the shoulder joint. Accordingly, it is becoming increasingly recognized that maladaptive plasticity [19, 20] expressed by overactivation [21] of different brain regions during the rehabilitation process represents an important problem.

The facilitation/inhibition dichotomy is therefore a fundamental concept in the development of state-of-the-art approaches in physiotherapy. One example of its application is the Constraint Induced Movement Therapy (CIMT) [22]. This technique refers to the contralateral upper limb constraint (the arm without neurologic deficit) in a sling (or more recently the mitt). The idea is to induce immobilization (evoking inhibition, as hypothesized in this paper) of the “good” arm to help recovery of the hemiparetic arm. Despite the clinical relevance of this method, its neurophysiological basis remains to be understood [23].

Based on the expectation that arm elevation facilitates and the restraint of upper limb inhibits control of motor action, we studied interhemispheric communication as a function of such inhibitory/facilitatory interactions during limb action, using fMRI to help answer this important question in motor physiology.

## 2. Methods

### 2.1. Participants

Seventeen healthy volunteers (10 Female/7 Male; Age: 43 ± 13.5 years; 17 right handed) according to the Edinburg Handedness scale [24] participated in this study. They reported no history of neurological or psychiatric disease and they were not taking medication. The experimental procedures were approved by the ethics committee of Faculty of Medicine, University of Coimbra, Portugal. All subjects gave written informed consent according to Declaration of Helsinki prior to their participation. 

### 2.2. Functional Magnetic Resonance Imaging (fMRI) Scanning

#### 2.2.1. Motor Paradigm

All participants underwent one structural magnetic resonance scan and two functional magnetic resonance (fMRI) scanning sessions: (1) the dominant upper limb was restrained while the nondominant upper limb performed arm elevation; (2) the opposite stimulation pattern was applied (nondominant restrained upper limb and dominant facilitation of arm elevation). Dominant and nondominant arms are equivalent in the sense that both arms were submitted to the same manipulations across the experiment. Accordingly, the experimental conditions across experimental runs of fMRI sessions were equivalent for the dominant arm and nondominant arm. The experimental design was symmetrical in the sense that movements were studied in a mirror-like manner, using the same simple motor tasks across runs.

#### 2.2.2. Sequence of Motor Paradigm

**Figure 1:**
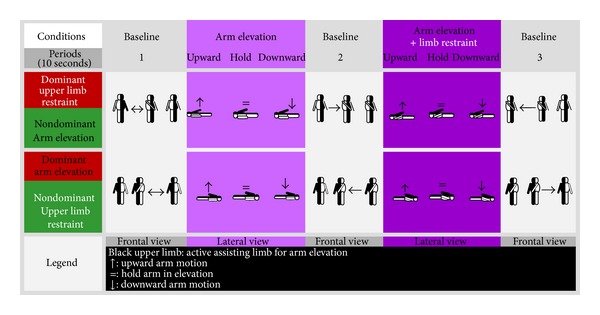
Schematic of experimental design in functional magnetic resonance imaging experiments. Limb manipulation during the experimental blocks (and control contralateral motion or restraint positioning during mid-period in baseline) is depicted by arrow symbols.

The sequence of each cycle of the motor paradigm was composed of five blocks (each lasting 30 seconds). The first, third, and the last block in a single experimental cycle were the baseline. Experimental conditions took place in between these blocks. The first condition consisted of a simple facilitation of arm elevation (AE). The second condition was combination of the facilitation of the arm elevation plus the contralateral upper limb restraining (AE + LR) to promote the inhibition of its motor action. All blocks were subdivided in three periods of ten seconds (see Figure 1). In total there were fifteen periods, repeated 10 times (cycle repetitions) with balanced experimental conditions. The schematic of experimental design in functional imaging experiments is described in Figure 1. To minimize the motion of the participant's head during the acquisition, a foam padding was employed. We also recorded the 6 parameters describing residual remaining head motion for the final correction, using a postacquisition standard motion correction method (run in Brain Voyager QX 2.4) for final coregistration of all acquired functional volumes.

#### 2.2.3. Detailed Task Description

**Figure 2:**
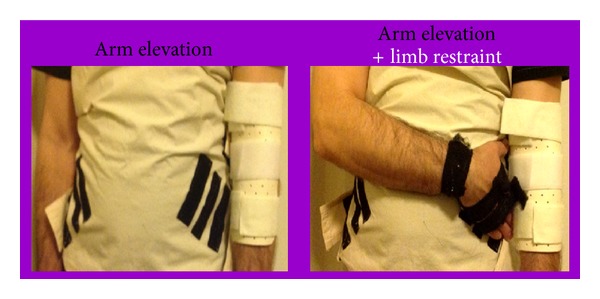
An illustration of the setup used to ensure restraint and specific mobilization of the shoulder joint.

Facilitation of arm elevation (AE) refers to the arm flexion, at the glenohumeral joint, with the elbow in full extension. A customized Cellacast splint was placed on the anterior part of arm and forearm in order to ensure elbow extension (Figure 2).

Near bore manual assistance by the researcher/physiotherapist was applied to all 17 subjects to help initialize arm motion and to orient the movement trajectory.

The facilitation of arm elevation was defined as a motor action composed by three periods with ten seconds each: upward, hold, and downward. The upward period was performed in order to promote concentric contraction of the flexor muscles of the shoulder. The hold period corresponded to the stopping of motion at 30–40 degrees (maximum range motion compatible with the space within of fMRI scanner) of the arm elevation and to the activation of the flexor muscles to sustain the upper limb, with isometric contraction. In the downward period, when the upper limb returns to the neutral position, the flexor muscles of shoulder had made the eccentric contraction. During all periods, shoulder elevation had one slow trajectory. To facilitate the movement, a mobilization was performed in assisting-active mode, in which the researcher/physiotherapist induced the movement. For each period, subjects heard verbal instructions indicating the motor activities and rest.

The baseline lasted 30 seconds. In the mid 10 seconds we applied or removed the restraint slings, as appropriate (Figure 2). The verbal instructions consisted of providing indications for “not move” in resting or “let move” in successive subphases.

The contralateral limb restraint (LR) was achieved by keeping shoulder adduction, crossing the arm in such a way that the hand was over the contralateral pelvis. Customized abdominal and hand slings with Velcro strips were used to ensure an efficient limb restriction and quick release.

We verified using MR compatible video monitoring whether restraint was effective. No mirror movements were observed during mobilization, ensuring that our procedure was sufficient to preclude movement.

#### 2.2.4. Data Acquisition

Magnetic resonance imaging data were collected on a 3 Tesla Siemens Tim Trio at the Portuguese Brain Imaging Network. High resolution anatomical images were acquired for each participant using a T1-weightd MPRAGE sequence 1 × 1 × 1 mm voxel size, repetition time (TR): 2300 ms, echo time (TE): 2,98 ms, flip angle (FA) 9°, field of view (FOV): 256 mm. The fMRI for each shoulder elevation (dominant and nondominant) was obtained using a T2 weighted BOLD contrast echo planar imaging sequence 2.5 × 2.5 × 3 mm voxel size, TR 3000 ms, TE 38 ms, FOV 256 mm. During each experiment, T1-weighted anatomical images were collected first followed by the functional runs. Each set included 10 continuous scans for first run and second run.

#### 2.2.5. Image Processing and Data Analysis

The imaging data analysis was performed using the Brain Voyager Software (QX version 2.4; Brain Innovation B.V., The Netherlands; http://www.brainvoyager.com). Before applying statistical analysis, several preprocessing approaches were performed. First, head motion was corrected (all <2 mm) and three-dimensional temporal filtering and slice scan time correction were performed. The head motion correction algorithm uses three translational (displacement) parameters and three rotational parameters. These six parameters are appropriate to characterize motion of rigid bodies: spatial displacement can be described by translation along the *x*-, *y*-, and *z*-axis and rotation around these axes. These parameters are estimated iteratively by computing how a source volume should be translated and rotated in order to better align with the reference volume. Maps were automatically registered into the standard Talairach space.

In the first level, data were analysed for each subject separately using general linear models (GLM) to identify significantly activated voxels. After model estimation, experimental contrasts derived from each participant were calculated and analysed individually. A second-level analysis with the total number of participants, using one-way repeated measures ANOVAs, was run. In the first stage, whole-volume GLMs were computed and corrected for temporal serial correlations, for subsequent group inferences. Each fMRI session with tasks for dominant and nondominant shoulder elevation was then processed separately, using a random effects analysis (RFX). This allowed inferring whether the observed results might be generalized to the population. Statistical maps were corrected for multiple comparisons using the false discovery rate (FDR) procedure for individual analysis in for BrainVoyager QX (2.4.), with *P* < 0.05 and group analysis with FDR < 0,05. Cluster-size thresholding allowed for the definition of volumes of interest (VOIs) in relation to defined Brodmann regions.

#### 2.2.6. Statistical Models for Region of Interest Analysis (ROIs)

In order to compare the recruitment of brain regions induced by the contrast presence versus absence of contralateral limb restraint during arm elevation ((AE + LR) versus (AE)), we computed the number of significant voxels in regions of interest (ROIs) corresponding to defined regions of sensory and motor cortex (Brodmann areas 1, 2, 3, 4, 5, 6, 7, and 8) and secondary somatosensory representation (Brodmann 40); for other details see Tables 1–4. For comparison we used, as stated above, the contrast analysis of (AE + LR) versus (AE), with FDR < 0,05, *P* (corrected) < 0,001.

## 3. Results

### 3.1. Dominant Arm Elevation Is Accompanied by Deactivation of Ipsilateral Hemisphere

**Figure 3:**
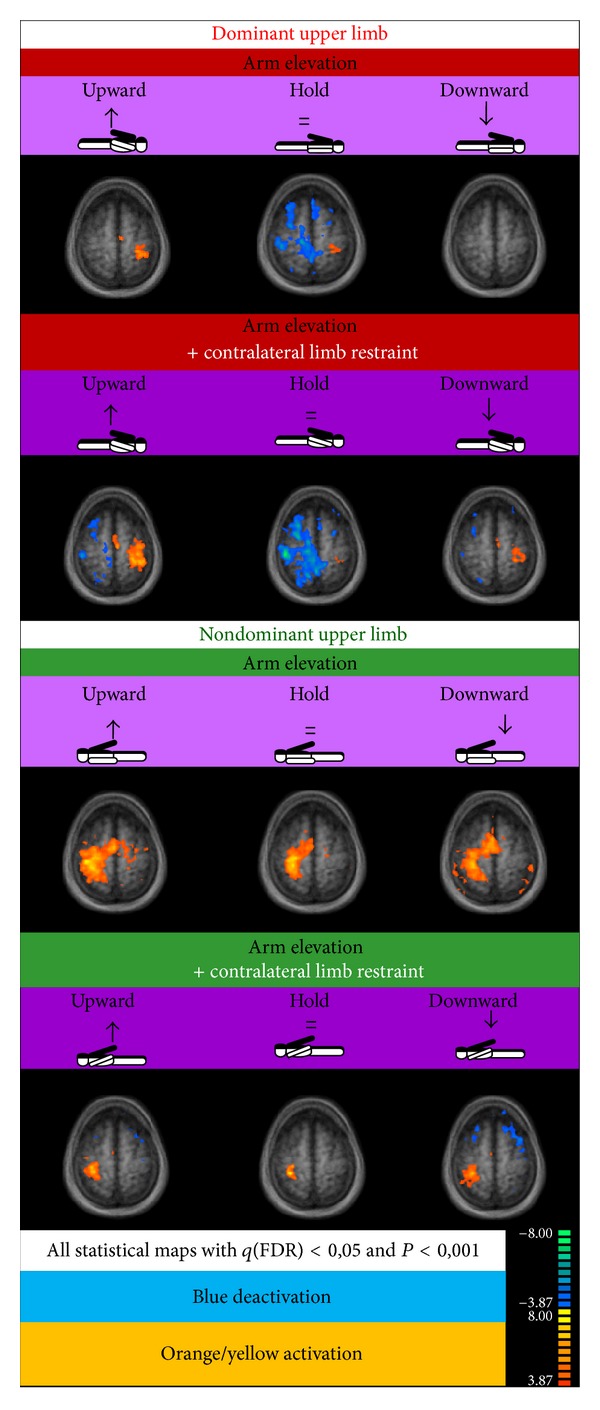
Statistical maps for tasks involving elevation coupled or not with restraint of dominant and nondominant upper limbs (*n* = 17, RFX, *P (corrected) < 0,001*).

Our results showed the expected activation of contralateral sensorimotor cortex and ipsilateral cerebellum during shoulder elevation. The dominant hemisphere elicited (as expected) less neural activation related to contralateral limb movement than the nondominant hemisphere (Figure 3). Importantly, we have found that only the dominant arm elevation was accompanied by statistically significant deactivation of ipsilateral sensorimotor cortex in particular during the “hold” period (see Figure 3). This signal reduction occurred without and with contralateral limb restraint but was augmented by the latter. In contrast, the nondominant limb showed no ipsilateral deactivation during limb elevation.

### 3.2. Contralateral Restraint Reduces the Area of Cortical Activation during Nondominant Limb Action, but Not for the Dominant Arm

We also found that dominant/nondominant limb action induced distinct cortical activation patterns in the presence/absence of restraint. Accordingly, contralateral restraint reduced the area of cortical activation during nondominant limb action, but not for the dominant arm. Tables 1, 2, 3, and 4 describe coordinates of brain regions that activate (orange) or deactivate (blue) as a function of dominant/nondominant limb action, with *P* (corrected) < 0,001.

## 4. Discussion

The main goal of this study was to understand how cortical interhemispheric interactions are modulated by motor facilitation versus restraint.

### 4.1. The Role of Hemispheric Expertise/Dominance

Our observations suggest a striking dissociation of deactivation versus enhancement patterns depending on hemispheric dominance. We found ipsilateral deactivation, in particular during the hold period, and contralateral enhancement, during the movement phase, for the dominant arm under restraint conditions. In contrast, specific ipsilateral deactivation was absent for the nondominant arm under such restraint manipulation.

In other words, dominant arm elevation elicited inhibition in the ipsilateral hemisphere (the one not planning movement). This already occurred without restriction but was enhanced by limb restraining. Nondominant arm elevation showed restriction induced smaller volume of significant activation in the contralateral (planning movement) hemisphere.

In summary, hemispheric dominance/expertise strongly influences the patterns of interhemispheric modulation induced by movement and restriction. Our result supports the hypothesis that interhemispheric control is asymmetric [25].

### 4.2. Ipsilateral Cortical Deactivation Only Occurs in Dominant Arm Elevation

The observed pattern of deactivation ipsilateral to the moving limb had been previously reported [26] but not the changes reported here concerning activation/deactivation imbalance with and without restriction and as a function of limb dominance/expertise. Our observations were most salient, as expected, during the hold phase of movement (arm elevation) corresponding to isometric muscle contractions.

Interestingly, our findings concerning the nondominant limb are also consistent with observations [5] that the nondominant limb often recruits larger volumes of activation in the motor cortex and surrounding regions. The cited study [5] did not however impose any modulatory manipulation. Our restraint manipulation for elevation of the nondominant limb yielded a much smaller elevation induced volume of activation.

Our results have large implications in terms of the physiology of plasticity, rehabilitation, and the development of therapeutical protocols that attempt to correct activation/deactivation balance.

The findings presented here may be helpful in the refinement of protocols for stroke rehabilitation such as the ones based on transcranial magnetic stimulation (TMS) [27–29]. This is because of the evidence shown here that modulation of deactivation/excitation is dependent on hemispheric dominance. Future studies should explore the efficacy of TMS protocols and/or physiotherapy of neurologic deficits involving the upper limb, based on these concepts.

### 4.3. Effects of Limb Restraint in the Absence of Contralateral Movement: Implications for Modulation of Rest Excitability

An isolated inhibitory effect of arm restraining was expressed by robust deactivation for contralateral hemisphere of nonmotor and motor areas.

This effect was therefore present not only during movement but also during rest. This suggests that, if tonically prolonged, it might possibly lead to long lasting structural changes. This was indeed observed by Langer et al. [30] who reported structural changes (decreases in cortical thickness) on ipsilateral primary motor and somatosensory area after 16 days of upper limb immobilization. Moreover, Avanzino et al. [31] refer to a reduction on activation on ipsilateral primary motor after 10 hours period of hand nonuse. Taken together, these observations may have real-life implications for rehabilitation in diseases such as stroke. Future longitudinal studies linking functional and structural changes directly will be helpful to support this idea.

### 4.4. Perspectives for Neurorehabilitation

The efficiency and monitoring of neurorehabilitation depends in part on the identification of the physiological effects [32] of available technical procedures and how they interact with the neurological condition being treated [33]. This is particularly relevant concerning the study of functional interactions (motor facilitation versus inhibition) for stroke rehabilitation [22, 34, 35].

### 4.5. General Implications and Future Work

Our results provide a physiological basis for interventions used in the physiotherapy domain, such as the inhibition technique known as the Constraint Induced Movement Therapy [22, 36]. This therapeutic approach is used in stroke treatment and our results suggest that different outcomes may be observed when applied to the nondominant or dominant upper limb.

For the first time we were able to induce in a quasinatural way modulation of brain activity taking advantage of the process of isolating and selecting each component of the full motor action in dorsal decubitus position. Our findings can potentially illuminate the adjustment of current protocols [37] in physiotherapy and TMS based on the support for inhibition theories provided by this work.

fMRI proved to be a promising measure that might be used in the future for fine tuning of clinical decision making in neurorehabilitation, as a complementary tool to other technologies such as electroencephalography and TMS [38].

## 5. Conclusion

We have found that modulation of interhemispheric communication based on motor inhibition/facilitation is strongly dependent on hemispheric dominance/expertise. These findings have strong implications for the neurophysiology of neurorehabilitation.

## Figures and Tables

**Table 1:** Activated brain regions by isolated dominant arm elevation and combined dominant arm elevation with nondominant upper limb restriction.

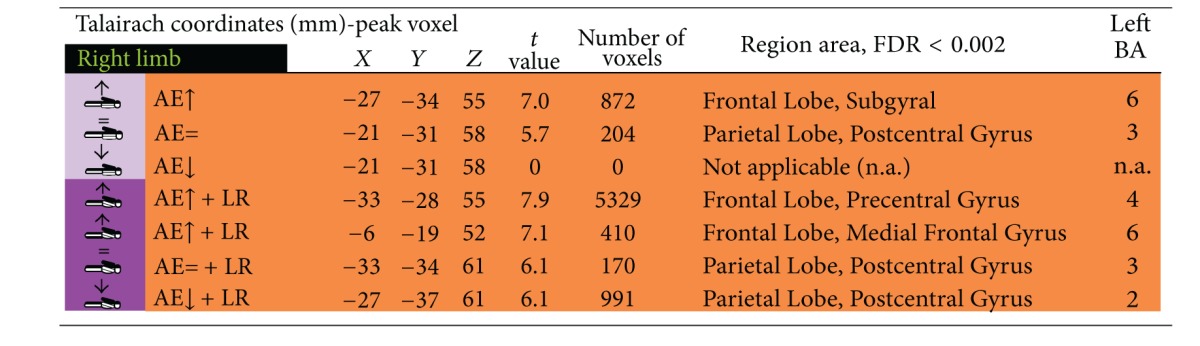

**Table 2:** Deactivated brain regions by isolated dominant arm elevation or combined dominant arm elevation with nondominant upper limb restriction.

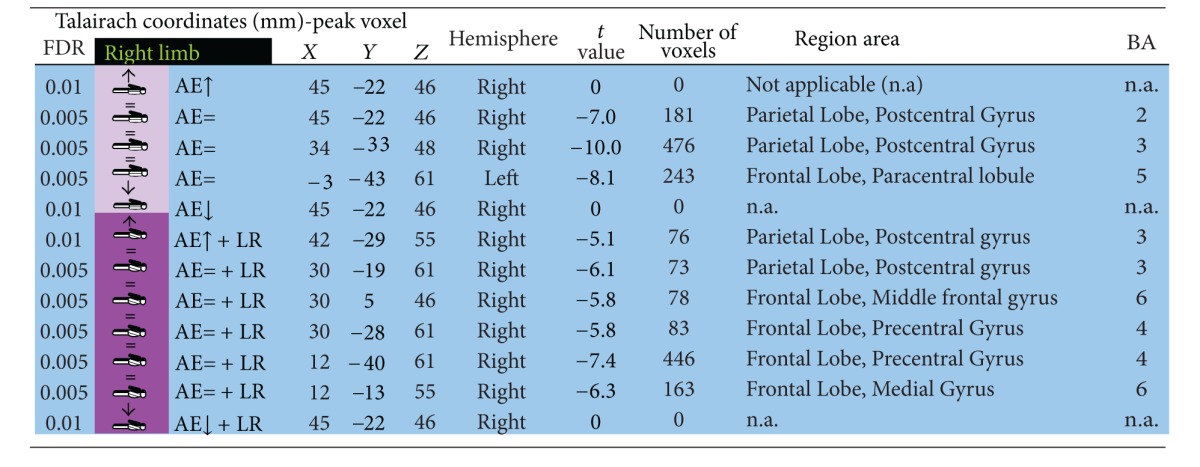

**Table 3:** Activated brain regions by isolated nondominant arm elevation or combined nondominant arm elevation with dominant upper limb restriction.

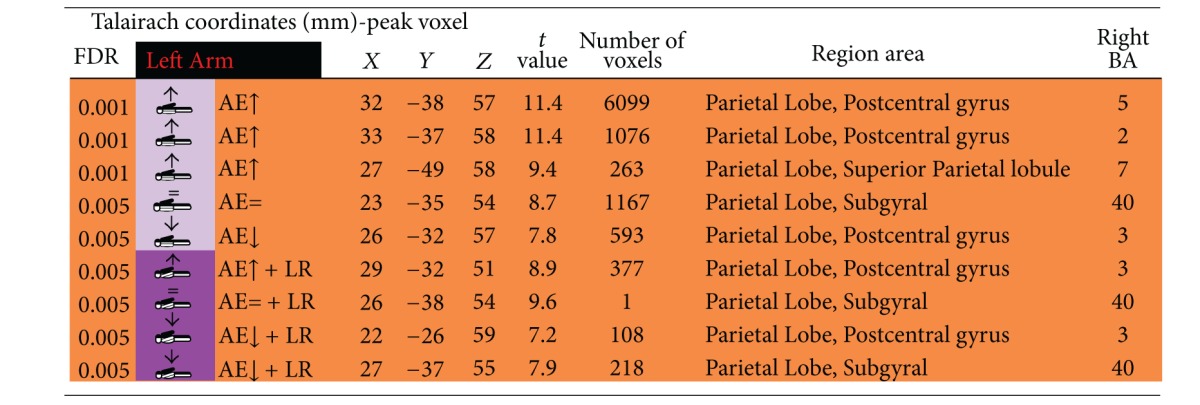

**Table 4:** Deactivated brain regions by isolated nondominant arm elevation or combined nondominant arm elevation with dominant upper limb restriction.

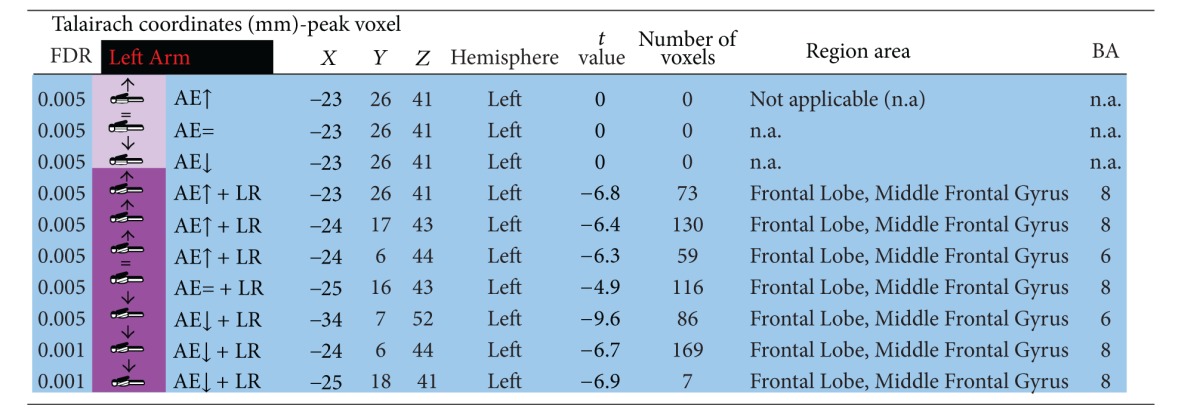

## References

[1] Chen R (2004). Interactions between inhibitory and excitatory circuits in the human motor cortex. *Experimental Brain Research*.

[2] Allison JD, Meador KJ, Loring DW, Figueroa RE, Wright JC (2000). Functional MRI cerebral activation and deactivation during finger movement. *Neurology*.

[3] Grefkes C, Eickhoff SB, Nowak DA, Dafotakis M, Fink GR (2008). Dynamic intra- and interhemispheric interactions during unilateral and bilateral hand movements assessed with fMRI and DCM. *NeuroImage*.

[4] Marchand WR, Lee JN, Thatcher JW, Thatcher GW, Jensen C, Starr J (2007). Motor deactivation in the human cortex and basal ganglia. *NeuroImage*.

[5] Singh LN, Higano S, Takahashi S (1998). Functional mr imaging of cortical activation of the cerebral hemispheres during motor tasks. *The American Journal of Neuroradiology*.

[6] Kocak M, Ulmer JL, Ugurel MS, Gaggl W, Prost RW (2009). Motor homunculus: passive mapping in healthy volunteers by using functional MR imaging—initial results. *Radiology*.

[7] Nirkko AC, Ozdoba C, Redmond SM (2001). Different ipsilateral representations for distal and proximal movements in the sensorimotor cortex: activation and deactivation patterns. *NeuroImage*.

[8] Schieber MH (2001). Constraints on somatotopic organization in the primary motor cortex. *Journal of Neurophysiology*.

[9] Jueptner M, Weiller C (1998). A review of differences between basal ganglia and cerebellar control of movements as revealed by functional imaging studies. *Brain*.

[10] Nitschke MF, Kleinschmidt A, Wessel K, Frahm J (1996). Somatotopic motor representation in the human anterior cerebellum. A high-resolution functional MRI study. *Brain*.

[11] Shmuel A, Augath M, Oeltermann A, Logothetis NK (2006). Negative functional MRI response correlates with decreases in neuronal activity in monkey visual area V1. *Nature Neuroscience*.

[12] Smith AT, Singh KD, Greenlee MW (2000). Attentional suppression of activity in the human visual cortex. *NeuroReport*.

[13] Pasley BN, Inglis BA, Freeman RD (2007). Analysis of oxygen metabolism implies a neural origin for the negative BOLD response in human visual cortex. *NeuroImage*.

[14] Liu Y, Shen H, Zhou Z, Hu D (2011). Sustained negative BOLD response in human fMRI finger tapping task. *PLoS ONE*.

[15] Shmuel A, Yacoub E, Pfeuffer J (2002). Sustained negative BOLD, blood flow and oxygen consumption response and its coupling to the positive response in the human brain. *Neuron*.

[16] Cirstea MC, Levin MF (2000). Compensatory strategies for reaching in stroke. *Brain*.

[17] Levin MF, Kleim JA, Wolf SL (2009). What do motor “recovery” and “compensationg” mean in patients following stroke?. *Neurorehabilitation and Neural Repair*.

[18] Michaelsen SM, Dannenbaum R, Levin MF (2006). Task-specific training with trunk restraint on arm recovery in stroke: randomized control trial. *Stroke*.

[19] Pascual-Leone A, Amedi A, Fregni F, Merabet LB (2005). The plastic human brain cortex. *Annual Review of Neuroscience*.

[20] Takeuchi N, Izumi S (2012). Maladaptive plasticity for motor recovery after stroke: mechanisms and approaches. *Neural Plasticity*.

[21] Buma FE, Lindeman E, Ramsey NF, Kwakkel G (2010). Functional neuroimaging studies of early upper limb recovery after stroke: a systematic review of the literature. *Neurorehabilitation and Neural Repair*.

[22] Wittenberg GF, Schaechter JD (2009). The neural basis of constraint-induced movement therapy. *Current Opinion in Neurology*.

[23] Nudo RJ (2003). Functional and structural plasticity in motor cortex: implications for stroke recovery. *Physical Medicine and Rehabilitation Clinics of North America*.

[24] Oldfield RC (1971). The assessment and analysis of handedness: the Edinburgh inventory. *Neuropsychologia*.

[25] Beaulé V, Tremblay S, Théoret H (2012). Interhemispheric control of unilateral movement. *Neural Plasticity*.

[26] Stefanovic B, Warnking JM, Pike GB (2004). Hemodynamic and metabolic responses to neuronal inhibition. *NeuroImage*.

[27] Butler AJ, Wolf SL (2007). Putting the brain on the map: use of transcranial magnetic stimulation to assess and induce cortical plasticity of upper-extremity movement. *Physical Therapy*.

[28] Cohen LG, Ziemann U, Chen R (1998). Studies of neuroplasticity with transcranial magnetic stimulation. *Journal of Clinical Neurophysiology*.

[29] Grefkes C, Fink GR (2011). Reorganization of cerebral networks after stroke: new insights from neuroimaging with connectivity approaches. *Brain*.

[30] Langer N, Hänggi J, Müller NA, Simmen HP, Jäncke L (2012). Effects of limb immobilization on brain plasticity. *Neurology*.

[31] Avanzino L, Bassolino M, Pozzo T, Bove M (2011). Use-dependent hemispheric balance. *Journal of Neuroscience*.

[32] Cramer SC, Sur M, Dobkin BH (2011). Harnessing neuroplasticity for clinical applications. *Brain*.

[33] Hachinski V, Donnan GA, Gorelick PB (2010). Stroke: working toward a prioritized world agenda. *International Journal of Stroke*.

[34] Grefkes C, Nowak DA, Wang LE, Dafotakis M, Eickhoff SB, Fink GR (2010). Modulating cortical connectivity in stroke patients by rTMS assessed with fMRI and dynamic causal modeling. *NeuroImage*.

[35] Ward NS, Cohen LG (2004). Mechanisms underlying recovery of motor function after stroke. *Archives of Neurology*.

[36] Sirtori V, Corbetta D, Moja L, Gatti R (2009). Constraint-induced movement therapy for upper extremities in stroke patients. *Cochrane Database of Systematic Reviews*.

[37] Serrien DJ, Ivry RB, Swinnen SP (2006). Dynamics of hemispheric specialization and integration in the context of motor control. *Nature Reviews Neuroscience*.

[38] Rossini PM, Dal Forno G (2004). Integrated technology for evaluation of brain function and neural plasticity. *Physical Medicine and Rehabilitation Clinics of North America*.

